# Resolving State-Specific Energy Flow in Metal Nanoclusters Using 2D Electronic Spectroscopy

**DOI:** 10.1021/acs.jpclett.6c00400

**Published:** 2026-03-10

**Authors:** Daniel J. Heintzelman, Kenneth L. Knappenberger

**Affiliations:** Department of Chemistry, 8082 Pennsylvania State University, University Park, Pennsylvania 16802, United States

## Abstract

Sub-to-few-nanometer gold nanoclusters exhibit a manifold of electronic states that result in nanocluster- and state-specific mechanisms of energy flow, which present new opportunities for developing photonic materials. Due to the spectral congestion of conventional ultrafast transient methods, mechanistic insights into energy flow are difficult to achieve for these systems. The use of two-dimensional electronic spectroscopy (2DES) to resolve electronic relaxation dynamics with state specificity for metal nanoclusters is described, along with prospects for future research. Excitation-detection frequency correlations inherent to 2D measurements resolve the electronic relaxation within specific gold superatom states. The state specificity of 2DES is extended to distinguish the influences of the electronic state symmetry on carrier relaxation using polarization-dependent measurements. Crosspeak-specific 2DES has also been used to distinguish sequential relaxation through nondegenerate electronic state manifolds of nanoclusters from the collective dynamics of metallic nanoparticles. These results demonstrate the power of 2DES for aiding the understanding of metal nanocluster photophysical properties.

## Graphical abstract

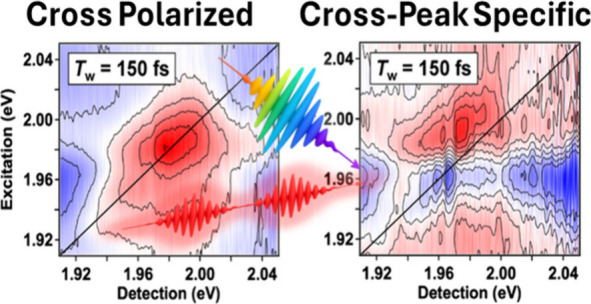

Noble-metal nanoclusters, confined to a few nanometers or smaller, provide novel opportunities to use and control energy flow.^1–5^ These nanoclusters are comprised of a dense manifold of unique electronic states, separated by energies spanning tens to hundreds of wavenumbers, and with properties determined by a number of factors, including quantum confinement, spin–orbit coupling, crystal-field splitting, and contributions from metal and ligand states.^6–11^ An advantage of ultrasmall nanoclusters is the ability tailor energy flow and optical properties through precision colloidal synthesis and isolation, which has resulted in structure-dependent spin-polarized emission, photothermal energy conversion, and nanoscale electronic-to-mechanical energy transfer.^12–18^ Ultrafast transient absorption spectroscopy (TAS) measurements have shown that nanocluster lattice structure and ligand choice can have dramatic influences over the rates of electronic energy relaxation in photoexcited systems.^19–24^ However, detailed state-specific mechanisms of energy flow are needed to fully capitalize on the unique attributes of ultrasmall metals. In this Perspective, recent advances in understanding state-specific mechanisms of nanocluster energy flow using two-dimensional electronic spectroscopy (2DES) are described, along with prospects for future breakthroughs in precision nanochemistry research.

Time-resolved TAS and 2DES are variations of pump–probe measurements that generate three types of transient signals: ground-state bleaching (GSB), excited-state absorption (ESA), and stimulated emission (SE).^25^ These three signal types report on complementary electronic pathways, thus making an overview of their origin helpful for interpreting transient data. GSB results from the depletion of ground-state electrons upon photoexcitation by the pump field, generating a negative-polarity TAS signal. Time-dependent GSB signals report on the global dynamics of the system and map the rates of carrier return to the ground state. ESA signals result from promotion of a pump-induced excited-state electron to a higher energy state by the probe pulse and produce a positive-polarity TAS. The time dependence of ESA signals reflects the population dynamics of specific excited states. SE signals are generated when the probe pulse stimulates the relaxation of excited carriers to the ground state and is accompanied by emission of a photon, generating a negative polarity TAS signal. Similar to ESA, SE tracks the dynamics of excited-state electrons.

Although the complementary nature of the three TAS signals described above provides opportunities to study state-specific dynamics in metal nanoclusters, practical challenges arise from the spectral gain bandwidth required to resolve ultrafast processes and the large density of nearly degenerate electronic states characteristic of metal nanoclusters. For example, a laser pulse with sufficient temporal resolution to resolve processes occurring on the sub-100 fs time scale requires a spectral bandwidth spanning several hundreds of wavenumbers, precluding resolution of signals produced by specific nanocluster states. The consequences of the mismatch between laser spectral bandwidth and the energy separation between nanocluster electronic states for studying energy flow is illustrated in Figure 1. The TAS spectrum generated by 400 nm excitation and visible probing of Au_25_(SC_8_H_9_)_18_^–^ are compared for multiple pump–probe time delays in Figure 1a.^23^ The TAS spectra are heavily congested, showing a progression of overlapping ESA peaks along with a negative-polarity feature at the approximate HOMO–LUMO energy gap of the nanocluster. Due to the large congestion of the 1D TAS spectrum, the negative-polarity feature is offset to positive TAS values at short pump–probe time delays because of spectral overlap with more intense TAS signals. This congestion makes the assignment of the negative-polarity signal difficult and the interpretation of time-domain signals problematic, often resulting in the use of global analysis methods for kinetic studies. The time-domain TAS signals resulting from different excitation conditions of Au_25_(SC_8_H_9_)_18_^–^ are plotted in Figure 1b and include a rapid (∼100 fs) decay, attributed to internal conversion, and a nondecaying component that persists beyond the measurement window.^23,26^ These kinetics are generally independent of the excitation conditions (wavelength and powers) employed across laboratories.^23^

**Figure 1:**
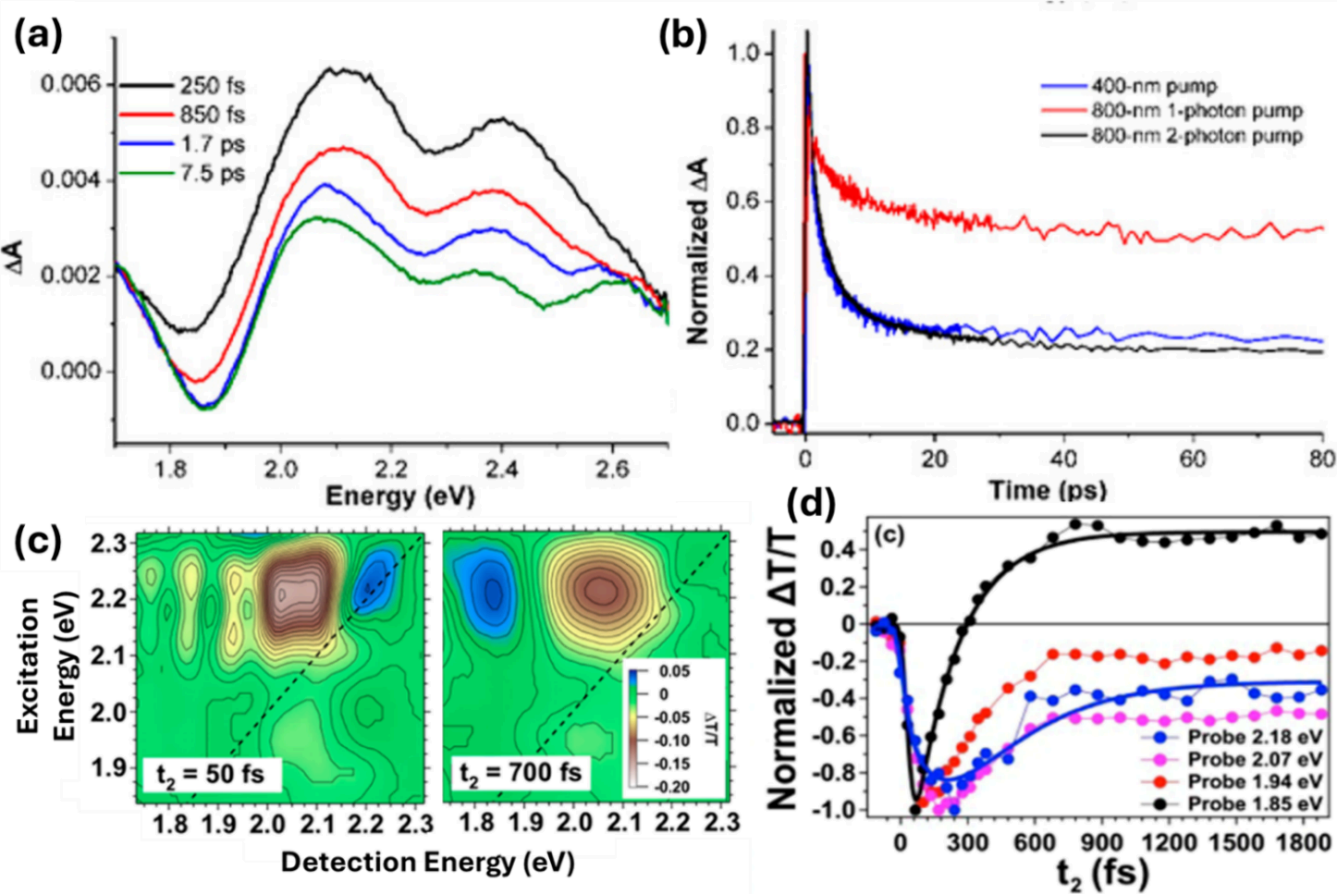
(a) fs-TA transient spectra of the Au_25_(SC_8_H_9_)_18_^–^ MPC at select waiting times. (b) fs-TA signal amplitudes of Au_25_(SC_8_H_9_)_18_^–^. [Reproduced from ref 23. Copyright 2017, American Chemical Society.] (c) 2DES at selected waiting times for Au_25_(SC_8_H_9_)_18_^–^. (d) Time-domain signals from 2DES due to dynamics of Au_25_(SC_8_H_9_)_18_^–^ superatom states following 2.20 eV excitation. [Reproduced from ref 26. Copyright 2016, American Chemical Society.]

The spectral congestion intrinsic to ultrafast TAS measurements of colloidal nanoclusters can be largely circumvented through the use of excitation-detection correlation plots used in 2DES.^27,28^ The 2DES technique used here is a third-order method implemented similarly to TAS that leverages four electronic field-sample interactions.^28,29^ A phase-locked pump-pulse pair, separated by an interpulse time delay called *t*_1_ interacts with the sample and allows for an excitation axis to be generated by Fourier transformation of the t_1_-dependent signal into the frequency domain. A second set of pulses (the probe and local oscillator) interacts with the sample at a waiting time called *T*_w_ or *T*_2_, which tracks the population dynamics of the system. The detection axis is generated by temporally delaying the probe and local oscillator by an interpulse time delay (*t*_3_), and subsequent Fourier transformation. Often, the probe is directly dispersed onto a detector and thus the local oscillator and probe propagate collinearly, and a Fourier transform of the final two fields is created in the detector Fourier plane.^30^

Examples of 2DES applied to Au_25_(SC_8_H_9_)_18_^–^ is shown in Figures 1c and 1d.^26^ 2DES maps recorded at *t*_2_ values of 50 and 700 fs are shown in Figure 1c. The map recorded at 50 fs shows a negative amplitude (blue) signal correlated along the diagonal at 2.2 eV and several ESA (yellow/brown) peaks detected at multiple excitation/detection off-diagonal crosspeaks. The ESA crosspeaks are detected at energy separations spanning from a few to as much as 700 wavenumbers, which agrees well with theoretical predictions for zero-field splitting values of the excited superatom D manifold of Au_25_(SC_8_H_9_)_18_^–^.^3,31,32^ Therefore, the 2DES approach clearly allows for state-selective and detection measurements that would not be resolvable with 1D-TAS measurements.

The time dependence of the 2DES signals (Figure 1d) also reveals insights into Au_25_(SC_8_H_9_)_18_^–^ electronic relaxation dynamics that are not apparent in the simple kinetic traces resolved by the 1D-TAS data shown in Figure 1b.^23,26^ The negative polarity signal detected on the diagonal at 2.2 eV rapidly decays as the initially excited carriers relax within the superatom D manifold within tens of femtoseconds, reflecting ultrafast state-to-state dynamics. The time dependence of the ESA peaks shows a state-specific buildup followed by a subsequent decay. Similarly, the bleach signal detected at the HOMO–LUMO region requires approximately 100 fs to form, corresponding to the creation of a superatomic exciton formed from superatom D electrons and superatom P holes. The dynamics revealed from 2DES are reminiscent of those measured for semiconducting nanocrystals and distinct from the simple molecular-based descriptions of internal conversion suggested by TAS measurements. Together, the excitation-detection correlation and time-dependent transient signals provided by 2DES provide access to the complex state-to-state relaxation mechanisms of gold nanoclusters, which are obscured in the congested spectra of conventional time-domain measurements.

Another example where the use of 2DES was essential to understanding the importance of state specificity in energy flow is the electronic relaxation dynamics of the Au_38_(SC_6_H_13_)_24_ nanocluster.^33^ Following the excitation of a charge-transfer resonance at 1.98 eV, polarization-dependent 2DES was used to distinguish different relaxation pathways for excited states corresponding to distinct electronic state symmetries. Figure 2a shows 2DES maps measured at pump–probe waiting times *T*_w_ = 50 and 150 fs, which were acquired using a cross-polarized pulse sequence (E_X_, E_X_, E_Y_, E_Y_).^34^ The map shown in Figure 2a obtained at *T*_w_ = 50 fs consists of a transient bleach signal (red) correlated along the diagonal as well as signatures of negative-amplitude cross-peaks at off-diagonal excitation/detection frequency correlations. Thermalization within the charge-transfer resonance is depicted in the map acquired at *T*_w_ = 150 fs, as is evident by the formation of a more isotropic, rather than diagonally correlated, transient bleach feature.^34^ The evolution from a correlated to anticorrelated transient signal is generally reflective of vibrationally mediated carrier relaxation within an electronic state. However, more precise descriptions of relaxation dynamics can be obtained when polarization dependent two-dimensional electronic spectroscopy (p-2DES) is used to generate crosspeak-specific signals, *S*_cp_.^35–37^ *S*_cp_-specific polarization schemes suppress the stronger signals of on-diagonal peaks and thus make the cross-peak signals easier to detect and analyze. *S*_cp_ maps can be isolated in two ways: (1) through subtraction of copolarized signal from three times the cross-polarized signal or directly via the ⟨0,0,60,–60⟩ polarization scheme.^35,37^ Figure 2b shows S_cp_ maps obtained at *T*_w_ = 50 and 150 fs. A stark contrast is apparent in the _w_ = 150 fs maps shown in Figure 2a and 2b. Whereas the Figure 2a data shows a transient bleach, the *S*_cp_ maps in Figure 2b show a prominent positive-polarity (blue) signal detected for state-selective excitation using 1.96 eV. As explained in ref 34, the signals rendered in blue result from electronic excitation of states with e-type symmetry, and those in red are from states with a-type symmetry. The time dependencies of these signals are compared in Figure 2c. The a-state signals show an ultrafast signal buildup, because of exciton formation, and subsequent decay due to carrier relaxation, similar to that suggested by the thermalization of the 2DES maps in Figure 2a. However, the e-type symmetry states follow a more complicated pathway that involves state-to-state energy transfer and evolves over the time frame of a few hundred femtoseconds. The results suggest that energy flow can be controlled in ultrasmall nanoclusters by the symmetry of the excited states.^34^ This is distinct from the thermalization processes of the larger nanoparticles. The combination of cross-peak specific 2DES transient signals, polarization-dependent selection rules, and theoretical descriptions of electronic states provides a level of precision in describing state-dependent nanocluster energy flow that is not achievable from 1-D transient spectroscopy.

**Figure 2:**
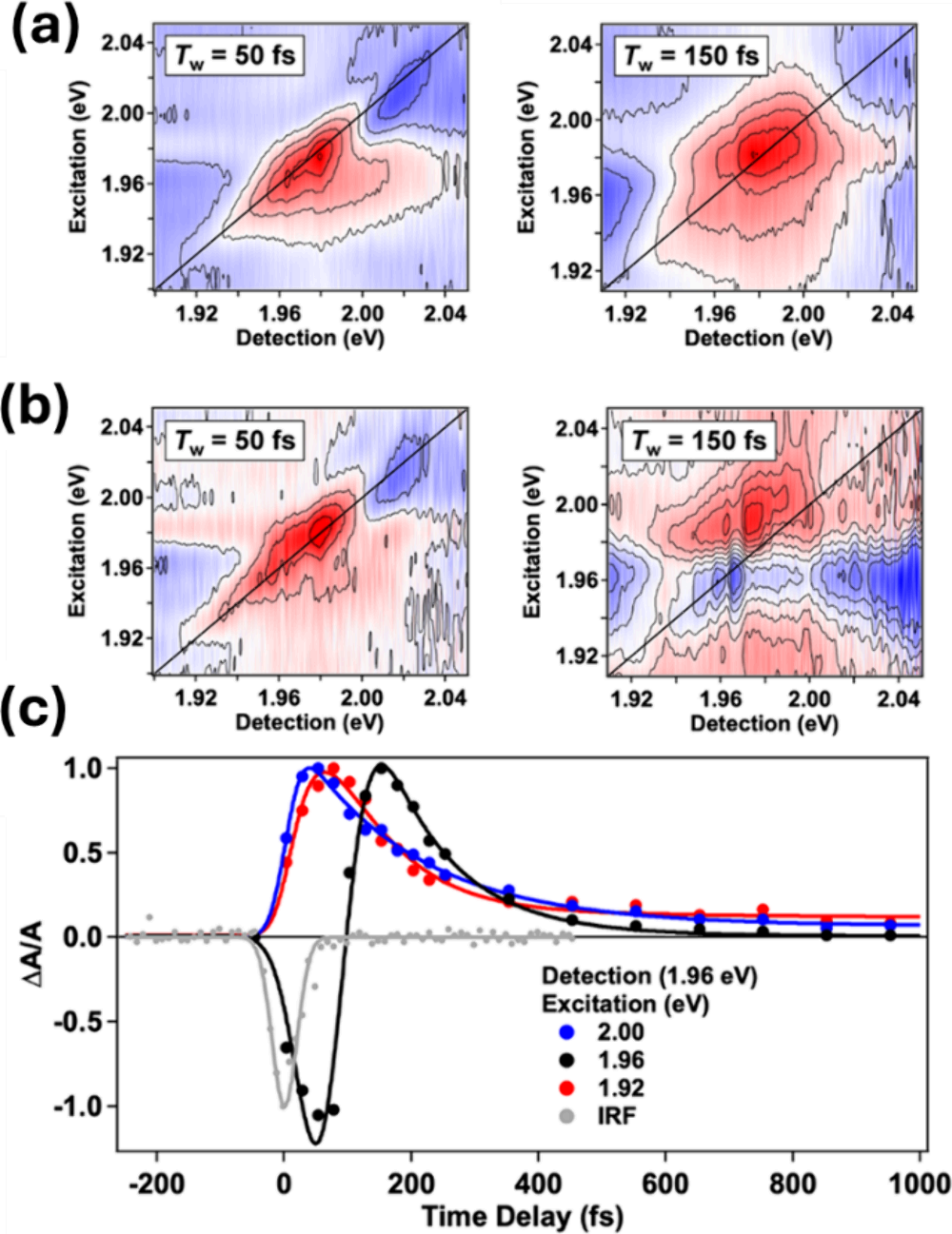
(a) Cross-polarized 2DES maps at two waiting times for Au_38_(SC_6_H_13_)_24_. (b) Crosspeak-specific, S_cp_, 2DES maps at two waiting times for Au_38_(SC_6_H_13_)_24_. (c) Time-dependent signal amplitudes from the S_cp_ 2DES maps at selected detection energies. [Reproduced from ref 34. Copyright 2023, American Chemical Society.]

p-2DES has also provided key insights for distinguishing between collective excitations of metals and noncollective excitations typical of gold nanoclusters.^38^ This is an important distinction because the nature of electronic excitation plays a determining role in the function of the nanocluster. Collective, phase-coherent plasmon excitations often mediate strong optical effects. As described above, the noncollective excitations typical of ultrasmall nanoclusters provide novel pathways for energy flow. An example of the success of p-2DES for distinguishing these excitations is the Au_42_(SC_8_H_9_)_32_ nanorod. The Au_42_(SC_8_H_9_)_32_ system supports a longitudinal resonance at 1.5 eV.^39–41^ Photoexcitation of this resonance yields an intense photothermal conversion of 27 °C after an excitation of 1 W/cm, which is competitive with leading photothermal transducers.^17^ This large response was initially attributed to a plasmon effect, which was surprising, given the small size of the nanorod.^17^ The electronic relaxation dynamics of this system was initially studied using transient absorption spectroscopy. Representative transient absorption spectra are shown in Figure 3a.^38^ The spectra in Figure 3a have been cut off between 13 200 and 13 500 cm^–1^ due to pump scatter obscuring the true line shape in this region. The TA spectra show a single bleach component at approximately 12 300 cm^–1^, which can be modeled with a normal distribution of energies, independent of the excitation pulse energy. The time dependence of these signals was also pulse-energy independent and exhibited an ultrafast decay (on the scale of hundreds of femtoseconds) and a nondecaying signal persisting on the picosecond time scale (Figure 3b). The detection of a single broad transient bleach at the longitudinal frequency is consistent with expectations for a metallic system. For metals, when excitation of a collective Fermi gas occurs, the carrier relaxation rates are expected to depend on the laser power following a quadratic dependence for ultrafast electron–electron scattering and a linear dependence for picosecond electron–phonon scattering.^22,42,43^ However, given the small size of these nanorods, excitation of a multicarrier electron gas may be difficult. As a result, the TA results do not provide conclusive information regarding the nature of the longitudinal resonance.

**Figure 3:**
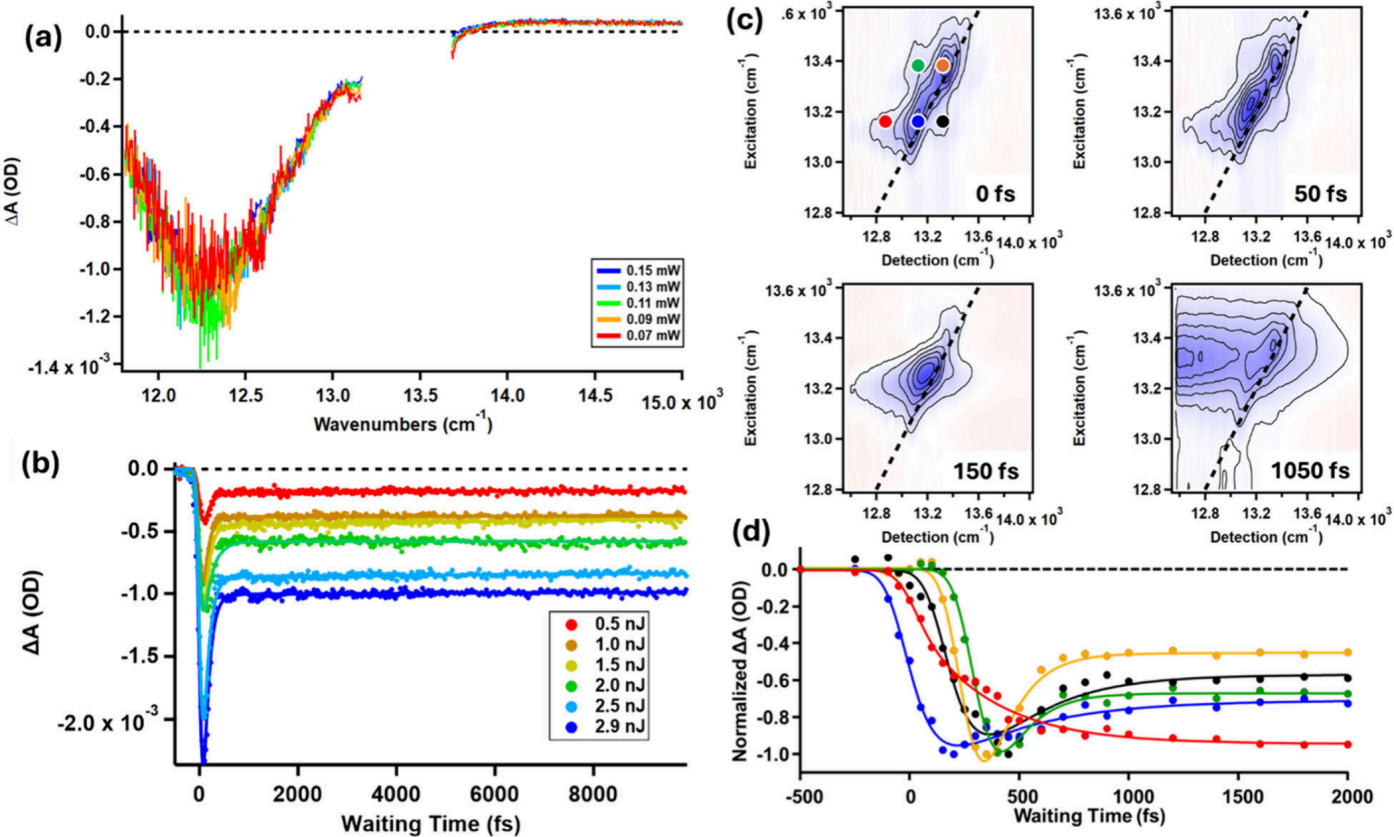
(a) Transient absorption spectra of Au_42_(SC_8_H_9_)_32_ from fs-TA compared for several excitation powers. (b) Time-domain transient absorption signals compared at several excitation pulse energies. (c) Au_42_(SC_8_H_9_)_32_ cross-peak specific, *S*_cp,_ 2DES maps at select waiting times. (d) Time-dependent signals from Au_42_(SC_8_H_9_)_32_ at select pump–probe coordinates (denoted by the dots in panel (c)) taken from the *S*_cp_ maps. [Reproduced from ref 38. Copyright 2025, American Chemical Society.]

In contrast to the TA results, the crosspeak-specific 2DES maps obtained from p-2DES shown in Figure 3c are rich with spectroscopic information.^38^ Several negative-amplitude signals, composed of bleach and stimulated emission signals, are detected along the diagonal as well as at off-diagonal crosspeak energies. The time-dependent amplitudes of these signals, shown in Figure 3d, evolve with peak specificity, indicating noncollective state-to-state energy flow rather than thermalization of a collective metallic excitation.

The case studies presented above provide a few key examples of how 2DES can be used to arrive at state-specific descriptions of energy flow in metal clusters. The prospects to employ 2DES methods for a deeper understanding of structure-dependent nanoscale energy flow are tremendous. The multidimensional approach provides numerous spectroscopic observables that have yet to be leveraged for metal nanocluster research. For example, time-dependent center-line-slope (CLS) analysis has been used to understand internal energy relaxation processes for molecules and colloidal nanoparticles.^44^ This approach could provide important insights into state-specific relaxation in metal clusters. In particular, the choice of passivating ligands and cluster-solvent interactions have all been shown to impact electronic relaxation rates in gold clusters.^14,19,45,46^ Applying CLS methods could reveal new information about how the design of cluster–ligand and cluster–solvent interfaces impacts energy flow.

Another area where multidimensional spectroscopy is poised to provide new insights is the coherent excitation of low-frequency vibrational modes in metal clusters. Figure 4a shows the oscillatory signals resulting from the excitation of 1-THz (blue) and 2.3-THz (red) modes in Au_144_(SC_8_H_9_)_60_ that were detected using transient absorption spectroscopy.^47^ Both signals were detected for a series of gold cluster sizes and assigned to coherent acoustic phonon modes. The 1-THz signal is assigned to a quadrupolar mode and exhibits a frequency dependence that scales with domain radius, consistent with expectations for an acoustic phonon.^47^ The 2.3-THz mode has been assigned to a breathing mode but shows a size-independent frequency when a cluster series is considered.^47–49^ Therefore, the precise origin of this vibrational signal remains open. More research will help to clarify the correct assignment of this signal.

**Figure 4:**
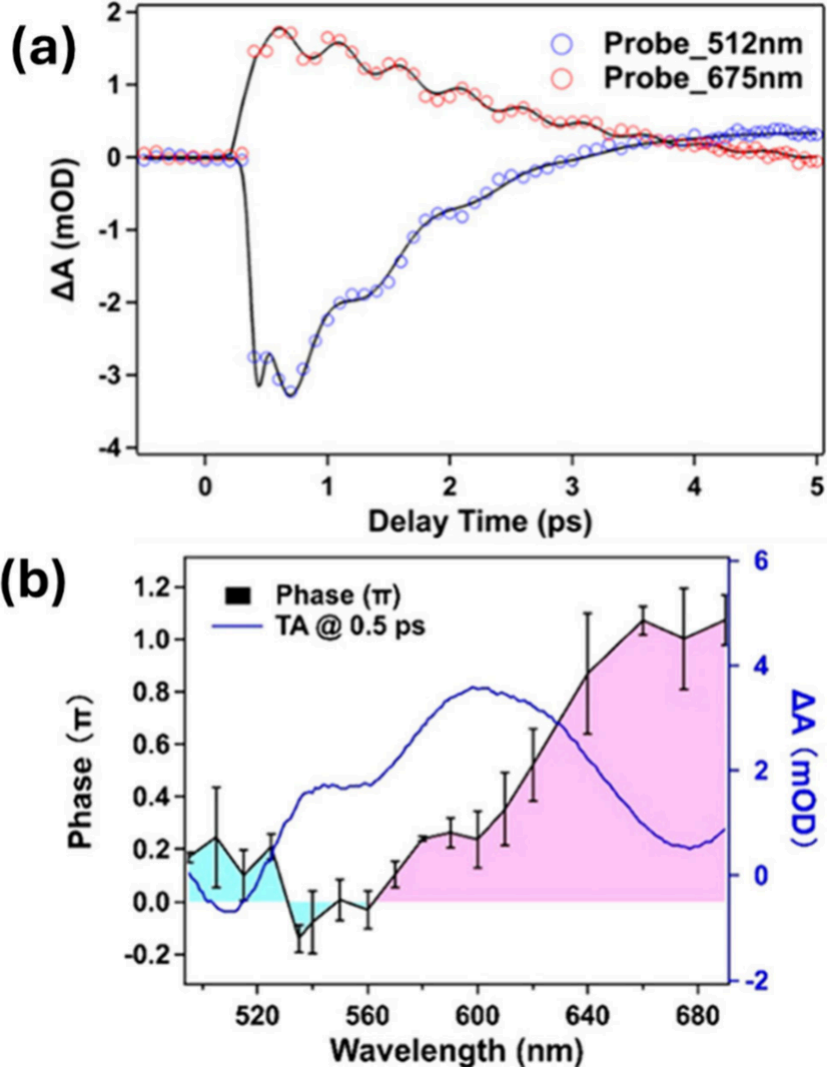
(a) Plot of the transient absorption signals detected at two wavelengths along with fits including coherent vibrational excitations. (b) Plot of the phase and transient signal as a function of detection wavelength for the coherent vibrations of Au_144_(SC_8_H_9_)_60_. [Reproduced from ref 47. Copyright 2023, American Chemical Society.]

An interesting open question involves the frequency-dependent phase detected for the coherent vibrational signals using visible TAS (Figure 4b).^47^ The observed phase shift is especially intriguing, because it may point to differences in the excitation mechanism of low-frequency vibrations for ultrasmall metal clusters. In the case of metallic nanoparticles, coherent modes are generally launched by an indirect mechanism, whereby ultrafast electronic cooling creates a “hot” lattice that subsequently induces low-frequency vibrational motion as an energy dissipation mechanism. The mechanism is indirect because of the mismatch in time scales between electronic cooling (approximately 100 fs) and the periods of low-frequency vibrations (picoseconds)—the electrons relax faster than a single vibrational period. A second, direct, mechanism for exciting acoustic vibrations exists.^48^ The direct mechanism occurs when the electronic lifetime and the vibrational periods are similar, as is the case for ultrasmall clusters that have electronic states spanning hundreds of femtoseconds, as highlighted in the above case studies, and short vibrational periods (500 fs) when compared with larger nanoparticles. The relative phase of the oscillations provides valuable information for distinguishing mechanisms that launch coherent vibrational dynamics, because the direct and indirect mechanisms are offset by a π-phase shift. The ability of multidimensional spectroscopy to provide an excitation axis would enable the study of coherent vibrational signals with both state-selective excitation and detection. This type of correlation would map vibrational excitation mechanisms with state specificity and provide direct insight into the interplay between electronic state lifetime and coherent phonon excitation. In addition to the fundamental information these insights would provide, they would also be useful for the design of nanoelectromechanical resonators (NEMS). Microscale electromechanical resonators are key components of integrated circuits. The predictive design of NEMS units would allow the miniaturization of device footprints but would require a detailed understanding of the state-specific energy flow. Multispectral phase analysis of coherent vibrational dynamics may also provide insights into state-specific energy transfer from “hot” carriers to low-frequency vibrations because the mechanism depends on the relative time scales of electronic states lifetimes and the periods of nuclear motion.

Several recent papers show that gold nanoclusters exhibit interesting electronic spin properties.^11,14,20^ In particular, spin polarized photoluminescence emission with degrees of spin polarization approaching 40% have been reported.^14^ Moreover, the observed spin polarization properties are heavily influenced by ligand passivation choice, but the mechanism of gold cluster–ligand interactions is not known. 2DES measurements that can resolve electronic spin dynamics will likely provide important insights for understanding why specific ligands are especially useful for increasing spin polarization. Similarly, multicolor 2D electronic vibrational measurements that can map electronic relaxation to ligand vibrational dynamics will be helpful for describing energy flow in these systems. The use of multipulse sequences with polarization control could provide significant advantages for isolating signals from spin-polarized electronic excitations. As predicted by Mukamel and co-workers, a sequence that employs (E_X_, E_X_, E_Y_, E_X_) polarized pulses should yield circularly polarized third-order responses.^50^ Alternating to (E_Y_, E_Y_, E_X_, E_Y_) would inverted the handedness of the nonlinear signals. Therefore, combining measurements using the appropriate optical fields with applied magnetic fields should isolate transient signals due to electronic spin.

In summary, the power of multidimensional spectroscopy for understanding energy flow in structurally precise metal nanoclusters has been described. Metal nanoclusters spanning from a few to a few hundred atoms benefit from synthetic precision, which allows chemists to tailor the energy flow. In large part, this control results from the strong interplay between the structure and state specificity of the energy flow. However, many key mechanistic details have been masked by conventional transient spectroscopy measurements. The multidimensional approach to ultrafast spectroscopy opens new opportunities to develop state- and structure-specific descriptions of energy flow in metal clusters. Some examples included in this Perspective are the possibility of leveraging superatoms for mediating energy flow and further refining these processes through electronic state symmetry. Next phases of research include opportunities for guiding electronic-to-mechanical energy flow through state-selective electronic excitation coupled with vibrational mode-selective energy transfer.
